# Environmental transmission of *Mycobacterium ulcerans* drives dynamics of Buruli ulcer in endemic regions of Cameroon

**DOI:** 10.1038/srep18055

**Published:** 2015-12-11

**Authors:** Andrés Garchitorena, Calistus N. Ngonghala, Gaëtan Texier, Jordi Landier, Sara Eyangoh, Matthew H. Bonds, Jean-François Guégan, Benjamin Roche

**Affiliations:** 1 UMR MIVEGEC 5290 CNRS - IRD - Université de Montpellier, Montpellier, France; 2 Ecole des Hautes Etudes en Santé Publique, Rennes, France; 3 Department of Global Health and Social Medicine, Harvard Medical School, Boston, MA 02115, USA; 4 Service d'épidémiologie et de santé publique, Centre Pasteur du Cameroun, Réseau International des Instituts Pasteur, Yaoundé, Cameroun; 5 UMR 912 - SESSTIM - INSERM/IRD/Aix-Marseille Université Faculté de Médecine, Marseille, France; 6 Unité d’Epidémiologie de Maladies Emergentes, Institut Pasteur, Paris, France; 7 Laboratoire de Mycobactériologie, Centre Pasteur du Cameroun, Réseau International des Instituts Pasteur, Yaoundé, Cameroun; 8 Department of Earth System Science, Stanford University, Stanford, CA 94305, USA; 9 UMMISCO, UMI IRD-UPMC 209, Bondy, France

## Abstract

Buruli Ulcer is a devastating skin disease caused by the pathogen *Mycobacterium ulcerans*. Emergence and distribution of Buruli ulcer cases is clearly linked to aquatic ecosystems, but the specific route of transmission of *M. ulcerans* to humans remains unclear. Relying on the most detailed field data in space and time on *M. ulcerans* and Buruli ulcer available today, we assess the relative contribution of two potential transmission routes –environmental and water bug transmission– to the dynamics of Buruli ulcer in two endemic regions of Cameroon. The temporal dynamics of Buruli ulcer incidence are explained by estimating rates of different routes of transmission in mathematical models. Independently, we also estimate statistical models of the different transmission pathways on the spatial distribution of Buruli ulcer. The results of these two independent approaches are corroborative and suggest that environmental transmission pathways explain the temporal and spatial patterns of Buruli ulcer in our endemic areas better than the water bug transmission.

Buruli Ulcer (BU) is a devastating skin disease caused by the environmental pathogen *Mycobacterium ulcerans (MU)* that mostly affects rural populations in Central and Western Africa^1^. Infection with *MU* and subsequent release of the toxin mycolactone by the pathogen causes extensive abrasion and necrosis of skin and soft tissues, muscle atrophy, and the formation of large ulcers^2^. Progression of the disease into severe stages, which is frequent in these regions due to limited access to treatment and the painless nature of initial lesions, is associated with a high disability burden, stigma and catastrophic costs for affected households^3^,^4^,^5^. The emergence of BU in these past 50 years as well as its geographic distribution have been widely associated with aquatic ecosystems especially in areas with slow-flowing and stagnant water^6^,^7^. However, the specific modes of transmission to humans are still poorly understood^1^. Plants, aquatic invertebrates and specific water conditions could allow *MU* to grow and persist in the environment^8^,^9^,^10^, pointing to two potential mechanisms of transmission –environmental and vector-borne– from aquatic sources.

The environmental transmission hypothesis suggests that *MU*, which is ubiquitous in endemic regions^8^,^11^,^12^, can be directly inoculated into the human dermis as a result of injuries taking place in contaminated environments, leading to infection^13^. This hypothesis is supported by epidemiological case-control studies where hygienic measures such as frequent baths, use of soap, and proper care of wounds all present significant protective effects^14^,^15^,^16^,^17^. Ecological studies in Africa have found *MU* in a wide range of environmental samples, such as soil, plants, water, aquatic macro-invertebrates and vertebrates, with concentrations ranging between 10^2^ and 10^4^ genome units/ml^8^,^11^,^18^,^19^. Experimental laboratory studies have shown that very few bacteria inoculated are sufficient to trigger BU-like lesions in mice (in the order of 10 to 10^3^)^20^,^21^. Alternatively, other authors have postulated the specific involvement of biting water bugs of the families Belostomatidae and Naucoridae (Order Hemiptera) as vectors of *MU* 22,23. Marsollier and collaborators demonstrated that *MU* is able to colonize the salivary glands of water bugs after feeding on contaminated prey, and can then be transmitted to mice through biting, leading to BU-like lesions after a few weeks^20^,^23^. Further support for this hypothesis is provided by the high concentrations of viable bacteria in water bug saliva^18^ and a protective immunological effect of exposure to water bug saliva^21^. Additionally, some case-control studies found a set of measures preventing insect bites to be also protective factors for the disease^16^,^17^. Nevertheless, field studies comparing water bug abundance and infection by *MU* in endemic and non-endemic regions have provided mixed results^18^,^19^,^24^.

Several characteristics of the disease limit to a great extent epidemiological and experimental studies, and are probably behind the confusion over BU risk factors and routes of transmission. The long incubation period of BU in humans^25^, combined with long delays in diagnosis among poor rural populations^26^, can cause recall bias in retrospective studies. In addition, the low annual incidence in endemic regions and the lack of a reliable serological marker for *MU* exposure limit the development of prospective cohorts. Experimental studies can provide important insights about specific mechanisms of transmission but they are restricted to controlled conditions that cannot inform about the relative importance of each transmission route in endemic areas. As previously demonstrated for other environmentally persistent pathogens^27^,^28^, it is possible that several transmission routes co-exist and contribute to the total burden of BU disease in humans. Understanding the relative contribution of each mode of transmission in endemic regions can inform appropriate indicators of disease risk and improve strategies for disease control and prevention. Thus, comparing the dynamics of the pathogen in the environment and water bugs with dynamics of the disease in humans can provide complementary insights. However, such an approach requires the use of high-resolution spatial and temporal data on *MU* and BU distribution from the same areas.

Thanks to an extensive environmental survey in Cameroon, the first systematic characterization of *MU* dynamics over space and time was performed in two BU endemic regions^11^. The objective of this study is to assess the relative contribution of each transmission route on the distribution of BU cases over space and time in endemic areas of Cameroon. For this, we integrate this extensive dataset of *MU* in the environment with human data on BU cases within the same regions (Fig. 1). We develop a transmission model to explain the temporal dynamics of BU incidence in Akonolinga district, Cameroon. We use this model to estimate rates of the two routes of transmission under a wide range of epidemiological and ecological parameters. Independently, we also estimate linear and additive statistical models to assess the relevance of the different transmission pathways to the spatial distribution of BU incidence. We find that the median force of infection for the environmental transmission in the best temporal predictions is 276 times larger than for the water bug transmission. These fits predict well the observed temporal patterns of BU (R^2^ = 0.6). Moreover, spatial distribution of BU is only associated with variables that are suggestive of environmental transmission. Both temporal and spatial approaches identify consistent shapes of the relationship between *MU* load in the environment and the temporal and spatial distribution of BU cases. The results of both independent methods are corroborative, each reinforcing the conclusion that environmental transmission pathways explain the temporal and spatial patterns of BU in our endemic areas better than the water bug transmission.

## Results

By simultaneously accounting for environmental and water bug transmission, we quantify the contribution of each mode of transmission to the temporal dynamics of BU cases in Akonolinga under a broad range of epidemiological and environmental parameters (Table 1). The best fit of mathematical models (AIC = 57.49) accurately predicted the monthly dynamics of observed BU cases (Fig. 2A) and suggested an exclusive role for environmental transmission (ratio λ_MU_/λ_WB_ larger than 10^3^). In addition, the mean force of infection in the set of 35 best fits that are considered equivalent (those with an AIC difference from best model lower than 2) was higher for the environmental transmission than for water bug transmission in 34 out of 35 fits. The ratio λ_MU_/λ_WB_, quantifying the importance of environmental transmission over water bug transmission, ranged from 0.86 to more than 10^6^, with a median value of 276 (Fig. 2B). Environmental transmission thus contributed to almost the entire burden of BU infections in our temporal model. The predictions from this set of best parameters had a high and significant correlation with the observed number of BU cases (Fig. 2C), with an R^2^ of 0.60 and 0.69 for incubation periods of 3 and 5 months respectively (these are the incubation periods present in the best models).

For the environmental transmission, the concentration of *MU* provided a better prediction of the temporal dynamics of the observed cases of BU than *MU* positivity. Indeed, fitted models based on *MU* concentration represented 100% of the total set of best 35 fits. A linear relationship between *MU* concentration and the force of infection, with a time of 6 months from infection to treatment, represented nearly two thirds of the fits. The mean AIC was lower in these fits than for fits with other times and functional links (Table 2). The mean value of this linear relationship is shown in Fig. 3A, along with the maximum and minimum values based on the parameters of the other best fits.

Our statistical models of *MU* spatial associations with BU incidence in both Akonolinga and Bankim indicate similar drivers as the mathematical model of temporal dynamics (Table 2). In the univariate linear models, environmental transmission was the only statistically significant predictor of BU incidence in populations within a 5km buffer. *MU* positivity in a water body was the best spatial predictor (R^2^ = 0.15, p-value<0.05), while *MU* concentration was only significant at the 90% level (R^2^ = 0.09, p-value<0.1). None of the variables for the water bug transmission showed any association with BU cases (R^2^ = 0.03, p-value = 0.77 for water bug positivity and R^2^ = 0.02, p-value = 0.36 for number of infected water bugs). Bivariate analyses in which one variable for water bug transmission was added at a time did not improve the model with the environmental transmission only.

We further explored whether a non-linear relationship between the *MU* environmental variables and BU incidence was more likely than a linear link by fitting GAMs of different spans. A GAM with a threshold effect and a saturation value significantly improved the results of the linear model for *MU* positivity (Table 2), but none of the nonlinear models improved the results for *MU* concentration. The predictions of the best statistical model are shown in Fig. 3B, along with their 95% confidence intervals (see Supplementary Materials, section S8 for a detailed description of all the statistical results).

In order to understand whether the spatial and temporal models provided the same information on the link between environmental *MU* and BU, we explored the relationship between *MU* positivity and *MU* concentration (Fig. 3C). The two variables were highly correlated, suggesting that both positivity and concentration provide similar information about *MU* environmental load (Spearman’s correlation test, p-value < 0.01 for both datasets). Furthermore, there was a clear plateau in *MU* concentration at higher *MU* positivity levels for both the temporal and spatial datasets. This suggests that once a positivity level was reached, this did not result in higher concentration in the samples. This would explain why we obtain a linear link with BU incidence for *MU* concentration but a saturation effect for *MU* positivity. We finally evaluated the strength of correlation between the temporal and spatial predictions for *MU* concentration only, and then for *MU* positivity. We found positive and significant correlations between the predictions of both models for each of these variables (Supplementary Materials, Section S6).

## Discussion

Buruli ulcer is one of the neglected tropical diseases in the world for which the mode(s) of transmission continues to be unclear, and for which there remains considerable debate in the scientific literature^1^,^13^. While experimental studies have shown that transmission through direct inoculation and water bug bites are both possible under laboratory conditions^18^,^20^,^21^,^23^,^29^, the relative importance of each of these transmission routes to human populations where BU is endemic is not established. Furthermore, the low number of cases in endemic areas hampers the development of cohort studies while the long incubation period and time to seek treatment of the disease obscure the results of case-control studies. This calls for innovative epidemiological study designs. In this study, we compare *MU* spatial and temporal dynamics in aquatic ecosystems and water bugs with the dynamics of BU cases to determine the potential contribution of each of these routes of transmission to observed disease patterns. Our two different, independent, approaches reinforce the conclusion that *MU* is primarily transmitted directly from the environment. None of the analyses suggest that BU is transmitted primarily by water bugs despite the common prevalence of *MU* found in these insects^18^.

Environmental transmission – non-specific inoculation of *MU* from contaminated environments to humans–explains almost the entire observed temporal dynamics of BU incidence, whereas the contribution of the water bug transmission in our model is negligible. The fluctuations in environmental *MU* concentration over time explain the dynamics of BU cases in Akonolinga for the study period. However, the predictions from the best temporal fit seemed to reveal higher frequency variations in the number of cases over time than what was observed in the region (Fig. 2A). These differences are likely to be due to either seasonal changes in human exposure that are not taken into account in our model^30^ or to methodological differences in the estimation of the time-series for predicted and observed cases (Supplementary Materials, sections S2–S4). In any case, this disparity caused the model to over-predict by two cases in July and August and under-predict by two cases in November and December, and model residuals were normally distributed (Figure S10) suggesting random errors in the model predictions. Unfortunately, temporal fits for Akonolinga could not be tested in Bankim, as environmental data in this region was only collected every three months, which precludes the fitting of robust mathematical models. Future studies are thus needed to assess the generalizability of our temporal results to other endemic regions with diverse environmental conditions.

The time from infection to treatment in our best temporal fits was consistently 6 months. This implies that ecological processes that favour growth of MU in aquatic environments and boost its concentration at specific times of the year^9^ might result in an increase in human BU infections, on average, six months later. Nevertheless, individual variability around average values for the incubation period and time to seek treatment could influence the dynamics of the reconstructed time series and the relationships identified in the temporal model. In the absence of individual data for each patient’s incubation period and time to seek treatment for our 10-year time series, a reasonable approach is the use of average estimates for parametrization of deterministic models. Moreover, data from 2012 showed that the time to seek treatment for BU in Akonolinga hospital was relatively low thanks to active case finding from Médecins Sans Frontières in the region (median = 5 weeks; interquartile range = 3–12 weeks)^30^. Future extensions of this modelling approach could benefit from such individual information for the whole time series and from the use of stochastic models to assess the impact of variability in individual parameter values on model outcomes.

At the spatial level, we show that *MU* presence is associated with BU incidence even at the very local scale within endemic regions. Previous studies have linked *MU* positivity in the environment and water bugs with BU prevalence^8^,^18^, suggesting that *MU* positivity in water bugs can differentiate between endemic and non-endemic areas^18^, and that environmental *MU* positivity may predict BU prevalence spatially^8^. Other studies have explored the theoretical implications of these routes of transmission in mathematical models^31^. However, due to data limitations, no studies have considered the contribution of environmental pathways while controlling for potential water bug transmission or *vice versa*. Because we systematically collected samples over space and time from the whole aquatic community and from water bugs, we were able to study both transmission routes simultaneously. We show that in our study regions, the environmental presence and concentration of *MU* in a water body can better predict BU incidence in surrounding populations than any of the variables suggestive of water bug transmission. While *MU* concentration shows a clear linear relationship with BU incidence, the best models suggest a saturation effect for the link between *MU* positivity and BU incidence. Thus expansion of *MU* in the environment may not increase human infectious without simultaneous increases in concentration.

We use the positivity and concentration of *MU* in the whole community of aquatic organisms as proxies for direct environmental transmission, as they represent two ways of measuring spatio-temporal changes in *MU* environmental load. Although a diversity of samples has been used in the past to estimate *MU* environmental load, *i.e* water^8^,^32^,^33^, soil or mud^33^,^34^,^35^, aquatic plants^8^,^12^,^35^ and aquatic organisms^8^,^11^,^18^,^19^,^33^; it seems clear that communities of aquatic organisms are able to acquire *MU* from these multiple environmental matrices and transmit it through ecological networks to other aquatic organisms^13^,^36^. Aquatic communities are composed of organisms with multiple feeding strategies such as filter feeders that filter water, herbivores that consume aquatic plants, scavengers that eat detritus, and predators that consume other organisms, creating multiple pathways for infection when *MU* is present in any of those environmental matrices. We thus consider that estimation of *MU* prevalence and concentration in the community as a whole should provide a good representation of *MU* environmental load in these various matrices and can represent appropriate proxies for measuring the risk of human infection with *MU* contaminated environments.

Even though our results are clearly in favour of a higher contribution of environmental *MU* transmission, this should be taken with caution. A sensitivity analysis performed in the mathematical model shows that if the median time to seek treatment of BU patients was higher than the 1–4 month range that we initially considered based on our data, the water bug transmission could play a role, contributing up to 20% of the infections at specific times of the year in the best model fit (Supplementary Materials, section S7). Furthermore, estimates of *MU* prevalence in water bugs of the families Belostomatidae and Naucoridae are subject to some uncertainty, due to smaller sample sizes and the presence of other hemipteran families in some of the positive pools tested. Although most hemipteran families found in aquatic sites from endemic regions are capable of biting and have been found positive to *MU*^11^,^18^, their role in transmission is less studied. Similar ecological studies on *MU* transmission may consider overrepresentation of these two families when performing PCR testing, in order to decrease such uncertainty. Finally, other transmission routes such as aerosols and the role of mosquitoes as vectors of *MU* have been previously proposed and are not considered here^37^,^38^.

The extremely low incidence characteristic of the disease even in endemic regions (on the order of 2 cases/1,000/year in high risk areas), limits our ability to study predictors of BU indicence, since stochastic processes have a strong impact in the spatio-temporal dynamics of rare diseases^39^. Recent analyses of long time-series of BU cases in French Guiana^40^ and Cameroon^30^ suggest seasonal patterns in BU incidence and reveal persistent spatial clusters of cases over time that are associated with specific environmental traits^41^. This is consistent with ecological studies on *MU*, where similar spatio-temporal patterns are observed for the dynamics of the mycobacterium^11^,^42^. We benefit from these novel insights and attempt to overcome the issue of stochasticity in rare diseases by comparing our one-year environmental dataset with BU incidence in human populations from the previous 10 years. For this, we aggregate the human monthly data for the whole region, in order to obtain robust estimates for the temporal patterns, and then we aggregate the entire time-series at the spatial level, in order to obtain robust estimates for the geographical distribution of cases in the region. While an ideal approach would be to associate, for each point in time and space, *MU* in the environment with the respective BU incidence using stochastic models, the amount of data necessary to have reliable and sufficient estimates from environmental samples is not available today. Nevertheless, the present study uses one of the most exhaustive environmental datasets available today for *MU* research and reveals new and important insights on this mysterious disease.

The results presented here represent a first step towards integrative disease prevention and could largely improve early detection of BU cases. If the concentration of *MU* DNA in the aquatic environment can predict spatial and temporal risk of BU emergence at a very local scale, it may be possible to predict the risk of disease emergence through systematic environmental screening of *MU*. Such a strategy could be used in combination with epidemiological research to identify potential *MU* transmission hotspots prior to prospective studies, or to control for BU environmental hazard when evaluating behavioural or socio-economic factors in case-control studies. Unfortunately, due to the limited resources and poor infrastructure in BU endemic settings, it is very unlikely that the national public health systems could afford such a screening strategy for disease control, either technically or financially. Unless cheaper and easier tools for environmental screening of *MU* become available, prevention and early detection of BU cases is likely to benefit the most from a better understanding of the environmental drivers of *MU* presence and concentration^9^. Such insight could help identify environmental proxies of *MU* dynamics over space and time at local and regional scales, allowing the development of early warning systems which could improve disease control.

## Methodology

### Environmental and incidence data

Environmental data was collected as described in *Garchitorena et al. 2014*^11^. Briefly, between June 2012 and May 2013, periodic sampling of aquatic communities was performed monthly in 16 aquatic ecosystems across Akonolinga, a region of Cameroon where BU is endemic. To increase our spatial representation, samples were also performed every three months across a second endemic region, Bankim, in another 16 aquatic ecosystems. The same collection methods were systematically applied in each water body and all aquatic organisms collected were classified. *MU* detection and quantification was done through quantitative PCR (*IS2404* and KR sequences) in 3084 sample-pools of aquatic organisms belonging to the same taxonomic group and collected during the same month and same site (see Supplementary Materials, section S1). At least 6 sample-pools were tested for *MU* at each site and month, and more accurate estimates were available for a subset of sites in both regions every three months. This enabled the first systematic characterization of *MU* temporal and spatial dynamics in aquatic ecosystems of BU endemic areas.

Regarding the human data, in each of the Buruli ulcer endemic regions under study, the district hospital is the health facility responsible for the diagnosis and treatment of all Buruli ulcer cases in the region. Information on cases treated in these hospitals, available through the National Buruli ulcer Program, is a good indicator for estimating the spatial and temporal patterns of Buruli ulcer incidence in the region since it is based on active search of cases^6^,^30^,^41^,^43^. We used the database of BU patients treated at Akonolinga hospital from 2002 to 2012 (see Landier *et al.* 2014 for a detailed description)^41^ and treated at Bankim hospital from 2006 to 2011, since systematic recording of BU cases was set up later in this region^6^. Information such as village of residence and month of diagnosis were recorded. Population size was obtained for each village from the district hospital registries for community health activities^16^. These data were used to assess spatial and temporal trends in BU incidence at the village level.

### Mathematical model of Buruli ulcer temporal dynamics

**Figure 1:**
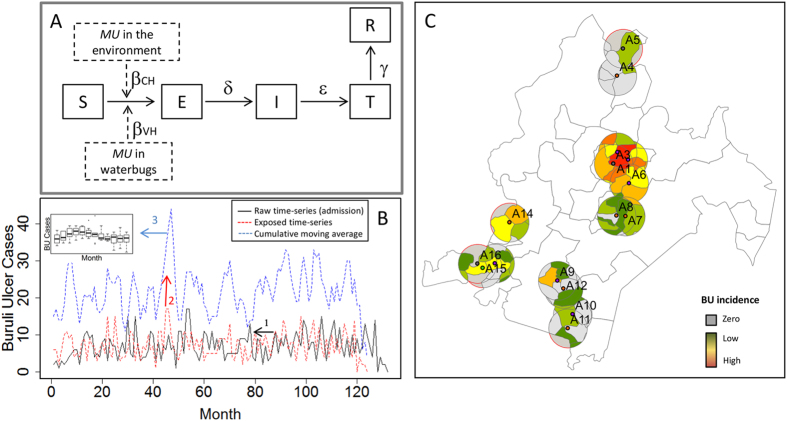
Temporal and spatial modeling of human Buruli ulcer cases in Akonolinga (Cameroon) from 2002 to 2012. (**A**) Framework of the mathematical (temporal) model. A susceptible individual (S) can be exposed (E) to BU from either the aquatic environment or water bugs, or both. After an incubation period 1/δ the individual will develop symptoms (I) and will get treated (T) after a time to seek treatment of 1/ε. Individuals recover (R) after a treatment time of 1/γ. (**B**) Temporal estimation of human cases. We first used the time-series of the monthly number of cases admitted to the hospital to estimate the time of infection according to different times to seek treatment and incubation periods. Secondly, we calculated the cumulative moving average of the infection time-series based on the incubation period. From this, we calculated the median and interquantile range for each month, which we used to fit the predictions of the mathematical model. (**C**) Spatial estimation of human cases. We calculated the cumulative incidence from 2002 to 2012 in 5km buffers around our sample sites. For this, the incidence of each village within the buffer was weighted according to the contribution of its surface to the total buffer surface. Map was generated using ArcGIS version 10.0 (ESRI Inc. Redlands, CA).

**Figure 2:**
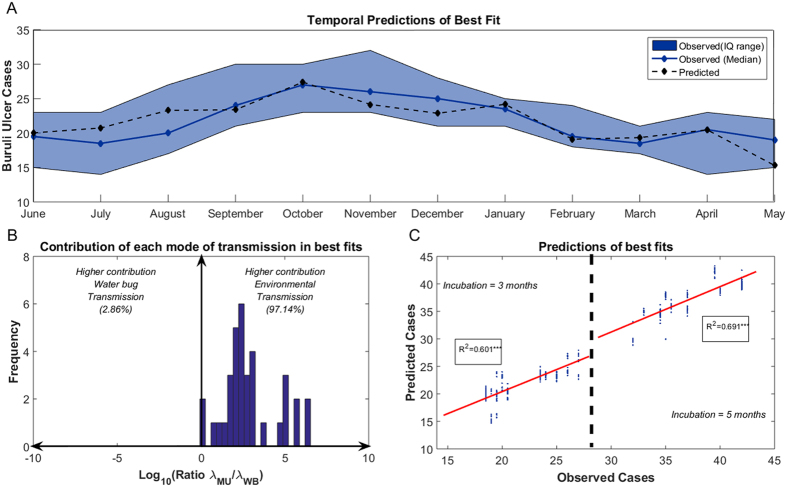
Predictions from the best fits in the mathematical (temporal) model. (**A**) Fitting from the best fit (AIC = 57.49). The solid blue line and blue patch represent the median value and the interquantile range for the number of Buruli ulcer cases per month estimated for model fitting. Dashed black lines represent the model predictions for each month. In this fit ε = 1/3, δ = 1/3, λ_MU _= 1.26E-4, λ_WB_ = 1.12E-7. (**B**) Ratio of the mean force of infection from the environmental transmission over that from the water bug transmission in the set of best fits. The ratio is in logarithmic scale (i.e. a ratio of 2 means that the environmental transmission was 100 times higher than the water bug transmission). The vertical blue bars represent the number of fits with a certain ratio λ_MU_/λ_WB_. (**C**) Predictions from the set of best fits (AIC = [57.49–59.49], n = 35) against the observed number of cases for an incubation period of 3 months (left) and 5 months (right). The solid red lines represent the linear regression line for each incubation period and the R-square of each regression is given in the inlet boxes (***p < 0.001).

**Figure 3:**
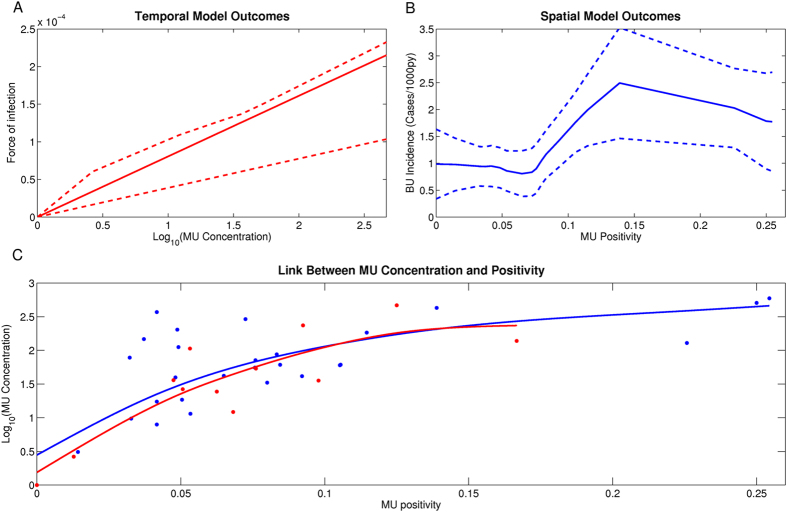
Relationship between *M. ulcerans* in the aquatic environment and Buruli ulcer in humans in the best temporal and spatial models. (**A**) Link between *M. ulcerans* and the force of infection for the environmental transmission in the best fit of the mathematical model (*MU* concentration). The solid line represents the mean value of the most represented functional form in the best set of fits and dashed lines represent the maximum and minimum values of force of infection for each value of concentration, based on all other functional forms in this set.(**B**) Link between *M. ulcerans* and Buruli ulcer incidence in the best spatial model (*MU* positivity). The solid line represents the predictions from the model and dashed lines are the 95% confidence intervals. (**C**) Link between *M. ulcerans* positivity and concentration in all sites (blue) and months (red). The dots represent the data and the solid line represents the fit using a smoothing spline.

A mathematical model was developed to link the temporal dynamics of *MU* and BU incidence in Akonolinga, Cameroon, since monthly environmental data was available for this region. In order to have robust estimates of the monthly dynamics of *MU* in the environment and in water bugs, monthly environmental data for Akonolinga was aggregated for the whole region (Supplementary Materials, section S1). We derive a Susceptible-Exposed-Infectious-Treated-Recovered (SEITR) compartmental model for the dynamics of BU transmission (Fig. 1A) that explicitly accounts for the epidemiological cycle of BU, notably the long incubation period and the delay in diagnosis. The goal of this mathematical model (fully described in Supplementary Materials, section S2) is to quantify the relative contribution of environmental transmission and water-bug transmission on BU dynamics via estimation of their respective transmission rates, *β*_*MU*_ and *β*_*WB*_. Thus, we determine these transmission rates under a broad range of epidemiological and environmental parameters (Table 1) to account for the uncertainty surrounding the epidemiology and transmission of the disease. Notably, we include multiple combinations of incubation period and time to seek treatment, different variables that are suggestive of environmental (*MU* concentration or *MU* positivity) or water-bug transmission (proportion or abundance of infected water bugs), different linear and non-linear relationships for the link between *MU* in the environment and the force of infection of the transmission route (to identify threshold or saturation effects), and multiple initial values for *β*_*MU*_ and *β*_*WB*_. The combination of all the parameter values that were tested represents a total of 7,200 different sets of parameters (see Supplementary Materials, section S3 for a detailed description of model simulations and fitting).

For each set of parameters, the model predictions for the monthly number of exposed are fitted to data on observed BU dynamics. For this, we use the 10-year admission data in Akonolinga hospital to reconstruct a time series of exposed individuals for each of the various combinations of incubation periods and times to seek treatment (Fig. 1B and Supplementary Materials, section S4). The fit performance was assessed by comparing the AIC values among the different simulations. The best fit was the simulation with the smallest AIC, and the set of fits with a difference of less than 2 AIC from the best model were considered equally performing. In these fits, we compare the force of infection from each transmission route, λ_MU_ and λ_WB_, to assess their contribution to the observed BU temporal patterns. All simulations were done with MATLAB, version R2013b.

### Statistical models for spatial associations

In order to complement the results from our temporal model by an independent approach, we studied the relationship between *MU* and BU incidence in both Akonolinga and Bankim from a spatial perspective using statistical models. First, spatial incidence of BU was estimated for 5 km circular buffers around each sample site (Fig. 1C and Supplementary Materials, section S5). We used linear models to identify which variables, suggestive of environmental transmission (*MU* concentration or *MU* positivity) or water bug transmission (proportion or abundance of infected water bugs), were statistically associated with the observed incidence around the sites. The values of these variables for each site were aggregated for the whole year in order to have robust estimates at each sample site in Akonolinga and Bankim (Supplementary Materials, section S1).

Secondly, we used generalized additive models (GAMs) to determine whether a non-linear model could better describe the relationship between the environmental presence of *MU* and BU incidence, *i.e.* threshold or saturation effects. The additive model uses a smoothing function to link the explanatory and response variables to characterize non-linear patterns in their relationship^44^. We fitted GAMs with LOESS smoothers of decreasing span and compared their performance based on their AIC values to determine the optimal span value^44^. Finally, the performance of all the models (linear and additive) was assessed through their AIC values and the model with the lowest AIC was selected. All statistical analyses were conducted with R software version 3.0.2 (R Development Core Team, R Foundation for Statistical Computing, Vienna, Austria), using the “base” and “gam” packages. Model assumptions were verified in the best performing models (Supplementary Materials, section S8). It is important to note that we refer to our statistical models of spatial associations as spatial models for simplicity, as a way to differentiate them from the temporal models. However, we do not claim to perform spatial modelling *per se*, as rigorous spatial modelling require the use of more complex frameworks that are not considered here.

## Additional Information

**How to cite this article**: Garchitorena, A. *et al.* Environmental transmission of *Mycobacterium ulcerans* drives dynamics of Buruli ulcer in endemic regions of Cameroon. *Sci. Rep.* **5**, 18055; doi: 10.1038/srep18055 (2015).

## Supplementary Material

Supplementary Information

## Figures and Tables

**Table 1:** List of parameters used in the mathematical model.

Symbol	Variable	Description	Value (Range)	Source
μ	Fertility/Mortality rate	New births/deaths per 1000 population per year	34.216	UN Population Division (Estimations for Cameroon in 2013)
1/σ	Incubation period	Time from infection to development of Buruli ulcer symptoms (months)	(3–5)	Uganda Buruli Group 1971;Veitch 1997; Lavender 2012;Trubiano 2013
*β*_MU_	Environmental transmission rate	Rate of direct transmission of *MU* from the aquatic environment to humans		Estimated from the models
*β*_WB_	Water bug transmission rate	Rate of transmission of *MU* from water bugs to humans		Estimated from the models
1/ε	Time to seek treatment	Time from development of Buruli ulcer symptoms to admission in the hospital (months)	(1–6)	Buruli ulcer Database (Own data)
1/γ	Time of treatment	Time from admission in the hospital to end of treatment (months)	(2–6)	Buruli ulcer Database (Own data)
*MU*_*pos*_	Total *MU* positivity in the environment	Total proportion of pools from all sites that were positive to *M. ulcerans* DNA	(0–0.17)	Environmental database (Own data)
*MU*_*conc*_	Mean *MU* concentration in the aquatic environment	Log10 of the mean concentration of *M. ulcerans* in pools from all sites (cfu/ml)	(0–3.31)	Environmental database (Own data)
*WB*_*pos*_	Water bug positivity to *MU*	Proportion of pools containing water bugs from the families Naucoridae and Belostomatidae that were positive to *M. ulcerans* DNA	(0–0.36)	Environmental database (Own data)
*WB*_*ab*_	Water bug abundance	Total number of hemipteran water bugs from the families Naucoridae and Belostomatidae collected from all sample sites	(150–599)	Environmental database (Own data)
*WB*_*inf*_	Total number of infected water bugs	Water bug positivity *Water bug abundance	(0–72)	Environmental database (Own data)

**Table 2:** Relationship between *M. ulcerans* in the aquatic environment and Buruli ulcer: summary of results for the best set of fits in the mathematical (temporal) model and for the statistical (spatial) models.

Mathematical Model						
Variable	Relationship	Time from Exposure to Treatment	Number of models	Mean λ_MU_	Mean λ_WB_	Mean AIC
***MUconc***	**Linear**	**6**	**26**	**1.27E-04**	**1.86E-06**	**58.52**
		7	5	1.26E-04	2.66E-07	59.33
		9	2	6.83E-05	6.25E-05	59.32
	Power law	6	2	1.29E-04	1.35E-07	59.32
**Statistical Models**						
**Model**	**Model Formula** 1	**Span (LOESS smoother)**	**a**	**b1**	**b2**	**AIC**
LM	y=a+b1*MUpos	–	0.82***	5.00*	–	78.96
	y=a+b1*MUpos + b2*WBpos	–	0.9***	5.60*	−1.26	79.98
	y=a+b1*MUpos + b2*WBinf	–	0.74 **	4.92*	0.01	80.07
**GAM**	**y=a+f(*****MUpos***)^2^	1	0.82	5.00	–	79.54
		0.9	0.82	5.00	–	77.52
		**0.8**	0.82*	5.00*	–	**75.55**
		0.7	0.82	5.00	-	75.92

*p-value < 0.05 ; ***p-value < 0.001.

Best mathematical and statistical model is represented in bold.

^1^Response variable (y) is the mean Buruli ulcer incidence in 5km buffers around the sites for the study period.

^2^The nature of the relationship using GAMs cannot be described with a single equation. See supplementary materials section S8 for a graphical description of the relationship for each value of span.
