# Supplementary material for: Environmental transmission of Mycobacterium ulcerans drives dynamics of Buruli ulcer in endemic regions of Cameroon
Source: Sci Rep. 2015 Dec 11;5:18055. doi: 10.1038/srep18055 (PMC4676024; doi:10.1038/srep18055)
Supplement: Supplementary Information [file srep18055-s1.pdf]

Environmental transmission of *Mycobacterium ulcerans* drives dynamics of Buruli ulcer in endemic regions of Cameroon
# - Supplementary Materials -

Andrés Garchitorena[1,2*], Calistus N. Ngonghala[3], Gaëtan Texier[4,5], Jordi Landier[4,6], Sarah Eyangoh[7], Matthew H. Bonds[3], Jean-François Guégan[1,2], Benjamin Roche[8]

[1] UMR MIVEGEC 5290 CNRS - IRD - Université de Montpellier I - Université de Montpellier II, Montpellier, France

[2] Ecole des Hautes Etudes en Santé Publique, Rennes, France

[3] Department of Global Health and Social Medicine, Harvard Medical School, Boston, MA 02115, USA

[4] Service d'épidémiologie et de santé publique, Centre Pasteur du Cameroun, Réseau International des Instituts Pasteur, Yaoundé, Cameroun.

[5] UMR 912 - SESSTIM - INSERM/IRD/Aix-Marseille Université Faculté de Médecine, Marseille, France

[6] Unité d'Epidémiologie de Maladies Emergentes, Institut Pasteur, Paris, France

[7] Laboratoire de Mycobactériologie, Centre Pasteur du Cameroun, Réseau International des Instituts Pasteur, Yaoundé, Cameroun

[8] UMMISCO, UMI IRD-UPMC 209, Bondy, France.

Section S1: Environmental data details

A. Molecular analyses for characterization of M. ulcerans DNA in environmental samples

Individuals collected from the same site and month were pooled together for PCR analysis by groups of aquatic organisms belonging to the same taxonomic group. Across the study, we tested at least 6 sample-pools for each month and each site in order to better describe spatio-temporal dynamics of *Mycobacterium ulcerans (MU)* presence and concentration. For this, we chose the 5 most abundant taxonomic groups in all sites, plus a sixth group that was different in each site, and we pooled all individuals of the same group. In addition, we chose 5 sites in each region, for which we applied a more in-depth molecular analysis every 3 months in order to have a better characterization of MU presence in taxonomic groups. For each sample-pool in the study, pooled individuals were all ground together and homogenized in 50 mM NaOH solution using Tissue Lyser II (QIAGEN). Tissue homogenates were heated at 95°C for 20 min. DNA from homogenized insect tissues was purified using QIAquick 96 PCR Purification Kit (QIAGEN), according to manufacturer's recommendations. 10% negative controls were included for extraction and purification.

Oligonucleotide primer and TaqMan probe sequences were selected from the GenBank IS*2404* sequence and the ketoreductase B (KR) domain of the mycolactone polyketide synthase (mls) gene from the plasmid pMUM001. QPCR mixtures contained 5 µl of template DNA, 0.3 µM concentration of each primer, 0.25 µM concentration of the probe, and Brilliant II QPCR master Mix Low Rox (Agilent Technologies) in a total volume of 25 µl. Amplification and detection were performed with Thermocycler (Chromo 4, Bio-Rad) using the following program: 1 cycle of 50°C for 2 min, 1 cycle of 95°C for 15 min, 40 cycles of 95°C for 15 s and 60°C for 1 min. DNA extracts were tested at least in duplicates and the 10% negative controls were included in each assay. Quantitative readout assays were set up, based on external standard curve with *MU* (strain 1G897) DNA serially diluted over 5 logs (from 10^6 to 10^2 U/ml). Samples were considered positive only if both the gene sequence encoding the ketoreductase B domain (KR) of the mycolactone polyketide synthase and IS*2404* sequence were detected, with threshold cycle (Ct) values strictly <35 cycles.

B. Estimation of temporal and spatial distribution of MU in the environment and water bugs

We used *MU* positivity (proportion of positive samples) and mean *MU* concentration in samples from the whole aquatic community as proxies for the environmental load of *MU*, which represents two ways of quantifying environmental transmission to humans. Aquatic communities are constituted of individuals of many functional groups, such as water filters, grazers of plants, scavengers eating detritus, predators of living insects among others, who can serve as hosts of *MU* in the aquatic environment[1,2] and therefore can reflect spatial and temporal changes of *MU* environmental load[3]. We used the decimal logarithm of *MU* environmental concentration instead of the raw mean number to account for the large variability in bacterial concentrations, which is common practice in microbiological studies. This also allows reducing the impact of potential measurement errors accumulated along the different laboratory steps of the qPCR analysis (extraction, purification, amplification), which are proportional to the initial concentration in the sample.

Regarding the variables suggestive of a water bug transmission, we used information from our dataset on water bugs from the families Belostomatidae and Naucoridae to estimate positivity of *MU* in water bugs and abundance of infected water bugs over space and time. Although other Hemiptera families have been positive to *MU* and are capable of biting[4], these two have been the most studied in laboratory experiments and have been proposed as vectors of the pathogen[4-8]. We estimated positivity to *MU* as the proportion of positive samples containing individuals from these two families (some samples also contained individuals from other Hemiptera families). Similarly, infections from a water bug could depend, not only on *MU* prevalence in the bugs, but also on the abundance of infected water bugs. We estimated this variable as the product of *MU* prevalence in these two families of water bugs and their total abundance, over space and time.

For each of these four variables, we first calculated the mean per month, using data from all sites in order to have robust estimates for the mathematical model of temporal dynamics (Figure S1). Then, we calculated the mean per site, using data from all months in order to have robust estimates for the statistical models of spatial associations.

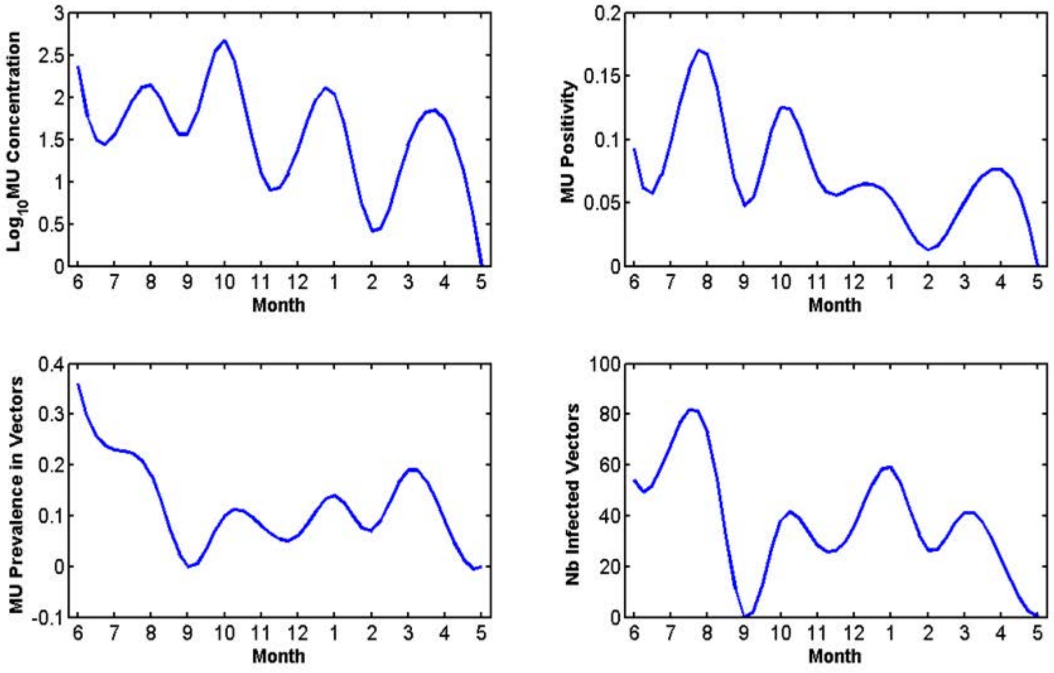

Figure S1. Monthly dynamics of environmental and vector variables used in the temporal model. Data on variables suggestive of environmental transmission (top) and water bug transmission (bottom) were aggregated for the whole region. Values thus represent the mean value at each month for all sites in Akonolinga, Cameroon. Data was collected from June 2012 (month 6) to May 2013 (month 5)

Section S2: Mathematical model framework

In this section, we explain the compartmental model we developed to link the temporal dynamics of *MU* and BU incidence in Akonolinga, Cameroon. The model is governed by the system of ordinary differential equations:

$$\frac{dS}{dt} = \mu N - \lambda_{MU}(t)\, S - \lambda_{WB}(t)\, S - \mu\, S \tag{1}$$

$$\frac{dE}{dt} = \lambda_{MU}(t)\, S + \lambda_{WB}(t)\, S - \sigma\, E - \mu\, E \tag{2}$$

$$\frac{dI}{dt} = \sigma\, E - \varepsilon\, I - \mu\, I \tag{3}$$

$$\frac{dT}{dt} = \varepsilon\, I - \gamma\, T - \mu\, T \tag{4}$$

$$\frac{dR}{dt} = \gamma\, T - \mu\, R \tag{5}$$

The total human population denoted by *N* is partitioned into four mutually disjoint classes representing disease status: susceptible (*S*) humans have the potential to contract *MU* but have not contracted *MU* yet; exposed (*E)* humans have contracted *MU* but do not show symptoms of the infection; infectious or clinically sick (*I*) humans exhibit visible symptoms of BU; and treated humans (*T*) are undergoing treatment until they recover (R). At the temporal and geographical scales we work, we assume that the human population is constant, so that the number of births matches the number of mortalities at each time[9].

The susceptible human class is populated by new births from all the classes (i.e., there is no vertical transmission of *MU*), which occur at rate μ, while humans from each class die naturally at the same rate. The host population move out of the susceptible class when they contract the infection either directly from the aquatic environment with force of infection λ_{MU} or indirectly from water bugs with force of infection λ_{WB}. Although we estimate a single and constant transmission rate for each route (β_{MU} and β_{WB}), temporal fluctuations in *M. ulcerans* in the environment, *MU(t),* and in water bugs, *WB(t),* induce fluctuations in the force of infection throughout the year. That is:

$$\lambda_{MU}(t) = \beta_{MU} f(MU(t)) \tag{6}$$

$$\lambda_{\mathrm{WB}}(t) = \beta_{WB} WB(t) \tag{7}$$

Susceptible hosts that have been infected from either of these routes increase the abundance of exposed class, which is then reduced when incubating hosts progress to the clinically sick state at rate σ. The size of the clinical host class is reduced when humans seek treatment at rate ε, while the treatment class lose humans when they recover at rate γ. We assume that all recovered patients become permanently immunised to the disease. Although it is still unclear whether *MU* confers immunity or not, or whether previously infectious BU patients can be re-infected after recovery, the 10- year database of BU patients used here was composed exclusively of new BU cases. Therefore, it is reasonable to assume in our modeling effort that there are no new infections after recovery.

Section S3: Mathematical model fitting and sensitivity analysis

In this section, we detail the sensitivity analysis we have conducted to test the robustness of our conclusions. For each set of parameters (see below), the model was run for 40 years to mimic the introduction of BU in human populations in Akonolinga, where cases have been reported since the late 60s[13]. Because we have only available data on 10 years of the BU program carried out by MSF (2002-2012), only the last 10 years in the simulation were used to calculate the median number of cases per month in the exposed class predicted by the model. These model predictions were then fitted to the median number of Buruli ulcer cases per month through maximization of the log likelihood (LL):

$$\mathrm{LL}= \ln\left(\prod_{j=1}^{N}\frac{1}{\sqrt{2\pi\sigma^2}}e^{-\frac{(x_j-z_j)^2}{2\sigma^2}}\right) = -\frac{1}{2}\left(N\,ln(2\pi) + N\,ln\,(\sigma^2) + \sum_{j=1}^{N}\frac{(x_j-z_j)}{\sigma^2}\right)$$

Where *N* is the number of data points (12 months), x_j and z_j are the predicted and observed number of cases for the month *j,* and σ^2 is the variance in the observed number of cases. From the log likelihood, an AIC was calculated for each model through:

$$AIC = 2\,n - 2\,LL$$

Where *n* is the number of parameters estimated from the model. Figure S2 shows a diagram explaining all the different combinations of parameters used in the sensitivity analysis. We test the relevance of both the mean concentration of *MU* in the environment (MU_{Conc}) and *MU* positivity (MU_{Pos}) as potential proxies of the environmental transmission. Regarding the water bug transmission, *MU* prevalence in water bugs (WB_{Pos}) and the number of infected water bugs (WB_{Inf}) are tested. In our simulations, we first assume a linear relationship between *MU* in the environment or water bugs and their respective force of infection.

$$\lambda_{MU}(t) = \beta_{MU} MU(t)$$

$$\lambda_{WB}(t) = \beta_{WB} WB(t)$$

Like many environmentally persistent human pathogens, a non-linear relationship could exist between the environmental presence or concentration of *MU* and the resulting force of infection of the environmental transmission[9,14,15]. For instance, some pathogens need to reach a certain abundance threshold or inoculum size to trigger human infections[14,15]. Others reach a certain saturation effect, meaning that a higher abundance of the pathogen does not result in higher number of infections[9]. For this reason, we test power and Gompertz functions to inform about the relationship between environmental *MU* and force of infection.

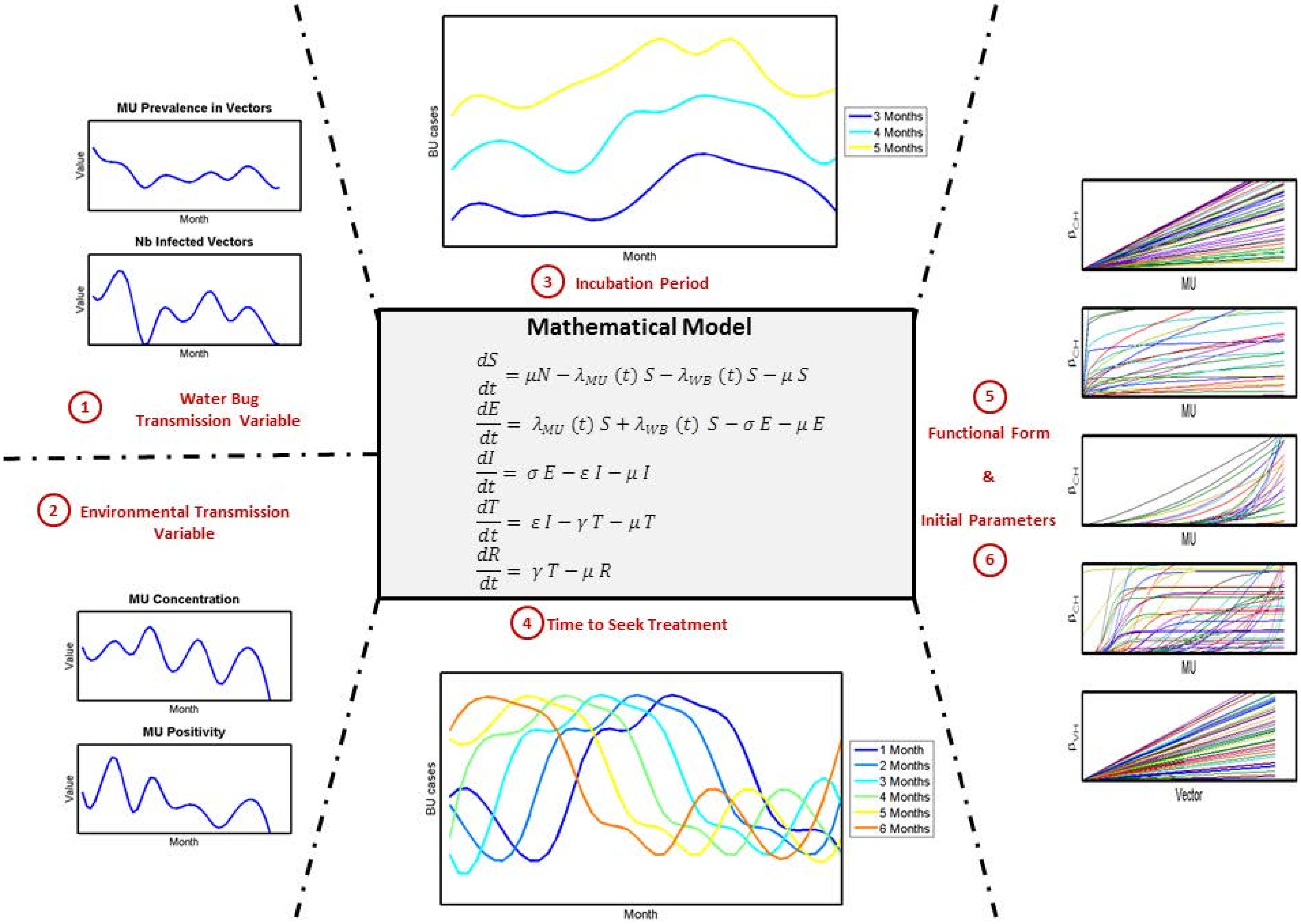

Figure S2: Sensitivity analysis carried out for the different parameters in the model. The predictions of the mathematical model, with framework outlined in the central box, is fitted to the dynamics of the observed cases for multiple combinations of 1) Water Bug and 2) Environmental transmission variables, 3) Incubation periods, 4) Times to seek treatment, 5) Linear and non-linear functional forms and 6) Initial parameters. As a result of this sensitivity analysis, 7,200 sets of initial parameters were fitted to the data.

The power function is described by:

$$\lambda_{MU}(t) = \beta_{MU} MU(t)^{a}$$

while the Gompertz function takes the following form:

$$\lambda_{MU}(t) = \beta_{MU}\, e^{-b\, e^{-c\, MU(t)}}$$

Therefore, for each mode of transmission, the dynamics of the two variables were used, all combinations of water bug and environmental variables were explored, and for each of the environmental variables, three functional forms were tested (see parameter ranges in Table S1).

Since we fit the *MU* dynamics to the BU time series, the model could be very sensitive to the estimated time to seek treatment in the population, which influences the lag between the two dynamics. Within an epidemiological study, the median time to seek treatment in Akonolinga was estimated at 2 months[16] so we initially considered a reasonable range of 1-4 months (these results are included in the main text) and then we explored the influence of considering longer times to seek treatment, such 5-6 months (these results are included in the section S7 of the supplementary materials).

In addition, the incubation period has an effect in our model, not only through the lag between the two dynamics but also because it affects the estimation of the cumulative number of cases (see section S4 for reconstruction of dynamics of exposed individuals used in model fitting). Longer incubation periods mean that people stay longer in the exposed group (before becoming infected) and therefore more people remain in the exposed group at a certain time. The incubation period was traditionally thought to be of about 3 months[17], but recent studies estimate it to be between 3 and 5 months[18,19], which is the range that we consider in our model.

Finally, for each combination of water bug variable, environmental variable, functional form of the environmental variable, time to seek treatment and incubation period, we performed a multiparametric sensitivity analysis by introducing 50 different initial values for the parameters estimated by the model, β_{MU}, β_{WB} (linear forms), *a, b* and *c* (non-linear forms). For each of the 50 simulations, we generated a random value within the maximum and minimum limits of each parameter. By doing this, we allocated different weights to each mode of transmission and we modelled different shapes of each functional form. The results of these multiple simulations, explained in the main text and section S6 of the supplementary materials, converge towards the idea that the environmental transmission contributes to most BU cases in our endemic region, regardless of the parameters considered or the initial values for each of the transmission rates.

Table S1. Range of parameters used to estimate force of infection λ for the different functional forms. Ranges were estimated for each water bug and environmental variable via visual evaluation to ensure that the dynamics of the predicted cases were within a realistic range and that a representative set of non-linear forms was tested for the power and Gompertz functions.

		WB Pos		WB Inf		MU Pos		MU Conc	
**Function**	**Parameter**	**Min**	**Max**	**Min**	**Max**	**Min**	**Max**	**Min**	**Max**
**Linear**	β	0	1.00E-03	0	5.50E-06	0	2.00E-03	0	1.50E-04
**Power**	β					0	4.00E-04	0	4.00E-04
	a					1	10	1	10
**Gompertz**	β					0	4.00E-04	0	4.00E-04
	b					-100	0	-100	0
	c					-100	0	-10	0

Section S4: Construction of times series of "exposed individuals" from admission data

In order to have robust estimations of the monthly dynamics of observed human cases, we aggregated monthly the human cases for the whole district. First, we calculated the monthly number of new cases in Akonolinga from 2002 to 2012. Then, we estimated the monthly time series of exposure, which happened several months earlier, by considering different incubation periods (3 to 5 months) and times to seek treatment (1 to 6 months). Because the model predicts the number of people present in a certain class at time *i,* and not the new number of individuals in that class, we estimated the cumulative number of exposed individuals from the time series of exposure based on the estimated incubation period as follows:

$$\text{Number of Exposed }(\text{Month}_i) = Admitted(Month_{i+j+k})$$

$$\text{Cumulative Number of Exposed }(\text{Month}_i) = \sum_{n=1}^{j} Exposed\left(Month_{i-(n-1)}\right)$$

Where *j* is the incubation period (in months) and *k* is the time to seek treatment (in months). This resulted in a time series of cumulative exposed individuals from 2002 to 2012 for each set of incubation period and time to seek treatment, which we used to calculate the median number of exposed individuals per month and the interquartile range in this 11 year period (Figure 1B). The first twelve months of the time-series were not used in these last calculations, since they represented the onset of the Buruli ulcer program in this region and the adaptive dynamics for the calculation of the cumulative number of cases.

Section S5: Calculation of spatially aggregated Buruli ulcer incidence

For each region under study, we aggregated data for the whole period and calculated the mean incidence of BU in 5km circular buffers around each site using ArcGIS version 10.0 (ESRI, Redlands, CA, USA) and R version 3.0.2[9]. For this, the mean BU incidence at each village contained within the buffer was calculated and weighted based on the contribution of the village surface to the total area of the buffer, and the incidence of the buffer was the sum of all weighted village incidences:

$$Incidence\ (buffer) = \sum_{i=1}^{n} \frac{Surface\ Village_{\ i} * Incidence\ Village_{\ i}}{Surface\ Buffer}$$

Where n is the number of villages within the buffer. This resulted in 31 sites for which we had information about *MU* presence and BU incidence in the 5km around it. This 5km value for the buffer was chosen because it represents a reasonable distance a person can walk to use a water body for professional or recreational activities in this region[11,12].

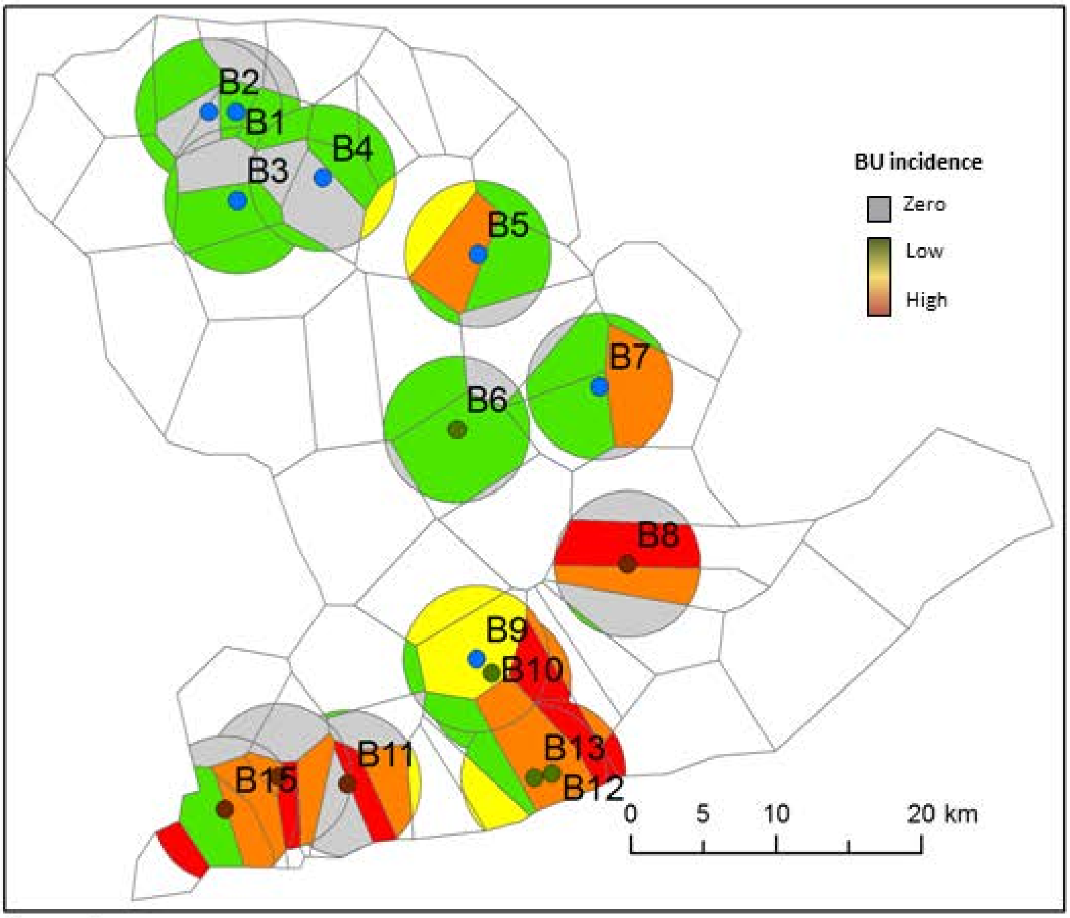

Figure S3 Spatial estimation of BU incidence in Bankim. We calculated the cumulative incidence from 2006 to 2011 in 5km buffers around our sample sites. For this, the incidence of each village within the buffer was weighted according to the contribution of its surface to the total buffer surface. Map was generated using ArcGIS version 10.0 (ESRI Inc. Redlands, CA).

Section S6: Correlation between temporal and spatial model results for each environmental variable

In the main text, we argue that the results for the best temporal model, with *MU* concentration (linear relationship), and those of the best spatial model, with *MU* positivity (sigmoid relationship), are consistent with each other given that there is a saturation in *MU* concentration at higher *MU* positivity levels. In this section we explain in detail the results of the best temporal and spatial model for each environmental variable separately, *MU* concentration and positivity. We show that for each environmental variable independently, the results of both models are consistent with each other, providing further support to our conclusions.

a) Models with MU concentration

MU concentration was the best predictor of BU temporal dynamics and there was a linear relationship with the force of infection (main text). However, the linear relationship in the spatial models was only significant at the 90% confidence level (Table S2). We explored if a non-linear relationship between *MU* concentration and BU was more likely than a linear link by fitting GAMs of different spans, but none of these models supported a non-linear link. The predicted slope of the linear relationship is shown in Figure S4A, along with the 95% confidence intervals. Finally, we compared the predictions from the spatial and temporal models and found a positive and significant correlation between the results of the two approaches (Figure S4C).

b) Models with MU positivity

The best fits for the temporal model with *MU* positivity had only power and sigmoid links (Table S2), which was consistent with the results for the spatial model, as we showed in the main text. These temporal fits were selected when considering fits within a 4 AIC difference from the best temporal model (medium support, otherwise none were selected). The mean value of the power law relationship is shown in Figure S4B, along with the maximum and minimum values. We finally compared the normalized predictions from both models for different values of *MU* positivity and found a positive and significant correlation between the results of both approaches (Figure S4D).

Table S2. Relationship between *M. ulcerans* in the aquatic environment and Buruli ulcer. Results for the best set of fits in the temporal model with MU positivity suggest a non-linear relationship with Buruli ulcer dynamics, while results for the spatial models with MU concentration suggest a linear relationship with Buruli ulcer distribution. This is consistent with the results presented in the main text.

Mathematical Models for *MU* positivity						
	Relationship	Time from Exposure to Treatment	Number of models	Mean λ_{MU}	Mean λ_{WB}	Mean AIC
MUpos	**Power law**	**6**	**20**	**1.29E-04**	**2.46E-06**	**60.78**
		7	5	1.23E-04	1.53E-06	60.55
**Statistical Models for *MU* concentration**						
**Model**	**Model Formula**	**Span (LOESS smoother)**	**a**	**b1**	**b2**	**AIC**
**LM**	**y=a+b1****MUcon***	**-**	**0.69 ***	**0.32**	**-**	**80.87**
	y=a+b1**MUcon* + b2**WBpos*	-	0.73 *	0.37	-1.21	82.04
	y=a+b1**MUcon*+b2**WBinf*	-	0.63	0.30	0.01	82.2
GAM	y=a+f(*MUcon*) [1]	1	0.69	0.32	-	81.86
		0.9	0.69	0.32	-	82.84
		0.8	0.69	0.32	-	82.96
		0.7	0.69	0.32	-	83.73

*p-value<0.05

Best mathematical and statistical model is represented in bold

[1] The nature of the relationship using GAMs cannot be described with a single equation. See supplementary materials section 4 for a graphical description of the relationship for each value of span.

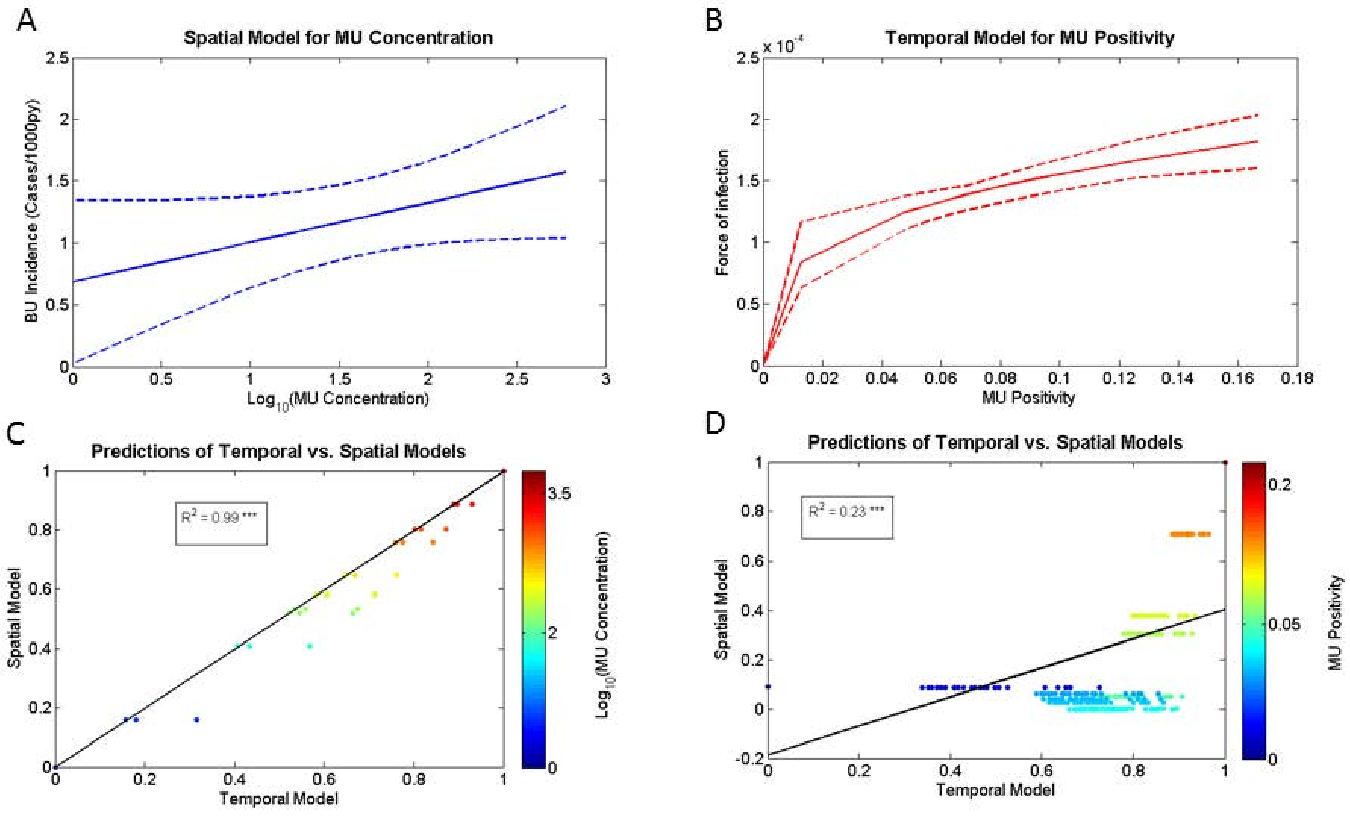

Figure S4. Correlation between temporal and spatial model results for each environmental variable, *MU* concentration and *MU* positivity.

A) Link between *M. ulcerans* and Buruli ulcer incidence in the best spatial model for MU concentration. The solid line represents the predictions from the model and dashed lines are the 95% confidence intervals.

B) Link between *M. ulcerans* and the force of infection from the environmental transmission in the best mathematical model fits for *MU* positivity. The solid line represents the mean value of the most represented functional form in the best set of models and dashed lines represent the maximum and minimum values of force of infection for each value of *MU* presence, based on all other functional forms.

C,D) Buruli ulcer incidence in the best spatial model is predicted for the *MU* values in the temporal models with *MU* concentration (C) and positivity (D), and the normalized predictions are compared to the normalized predictions from the set of best temporal models (***p<0.001).

Section S7: Results of the sensitivity analysis for longer times to seek treatment

Data on time to seek treatment of Buruli ulcer patients treated at Akonolinga hospital suggests that, despite a high individual variability, more than half of cases seek treatment within 2 months from realization of symptoms[16]. In the main text, we describe the results for an estimated time to seek treatment in the population of 1 to 4 months, which we believe is a reasonable range. Nevertheless, we tested the influence of increasing the expected time to seek treatment of the population beyond this range. When considering times to seek treatment of 5 and 6 months, slightly different results were obtained. The best fit, with a time from infection to treatment of 9 months, still showed a higher contribution for the environmental transmission, but the ratio $\lambda_{MU}/\lambda_{WB}$ was only 2.1. In this model, each of the two modes of transmission contributes differently to the temporal dynamics of BU cases (Figure S5), with the water bug transmission contributing to a significant number of infections (up to 20%) in the months of August and March and then decreasing the rest of the year. Results from the best set of fits are still in favour of the environmental transmission when considering times to seek treatment of 5 and 6 months, with 94% of best fits having a higher contribution of the environmental transmission (Figure S6), with a median ratio $\lambda_{MU}/\lambda_{WB}$ of 50. The lowest value of $\lambda_{MU}/\lambda_{WB}$ in this set of best fits is 0.97, which implies that the water bug transmission could contribute up to 51% of the total burden of disease.

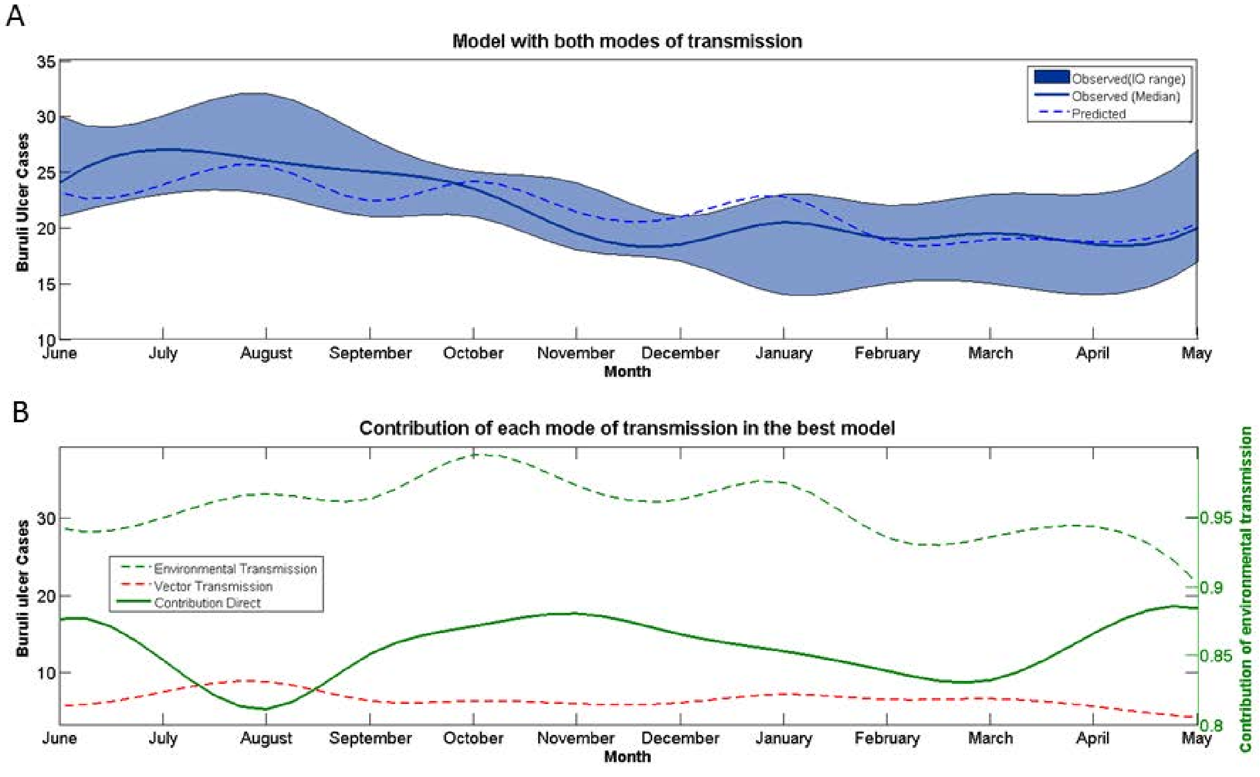

Figure S5. Predictions from the best fit in the mathematical model when considering times to seek treatment of 1-6 months.

A. Fitting from the best fit (AIC=56.86). The solid blue line and blue patch represent the median value and the interquantile range for the number of Buruli ulcer cases per month estimated from the 10-year times series of observed cases. Dashed blue lines represent the model predictions for each month. In this fit ε=1/6, δ=1/3, λ_{MU}=7.94E-5, λ_{WB}=3.75E-5

B. Contribution of each mode of transmission. The red and green dashed lines represent the number of cases per month predicted as a result of the vector and environmental transmission respectively. The solid green line represents the proportion of cases explained by the environmental transmission over time.

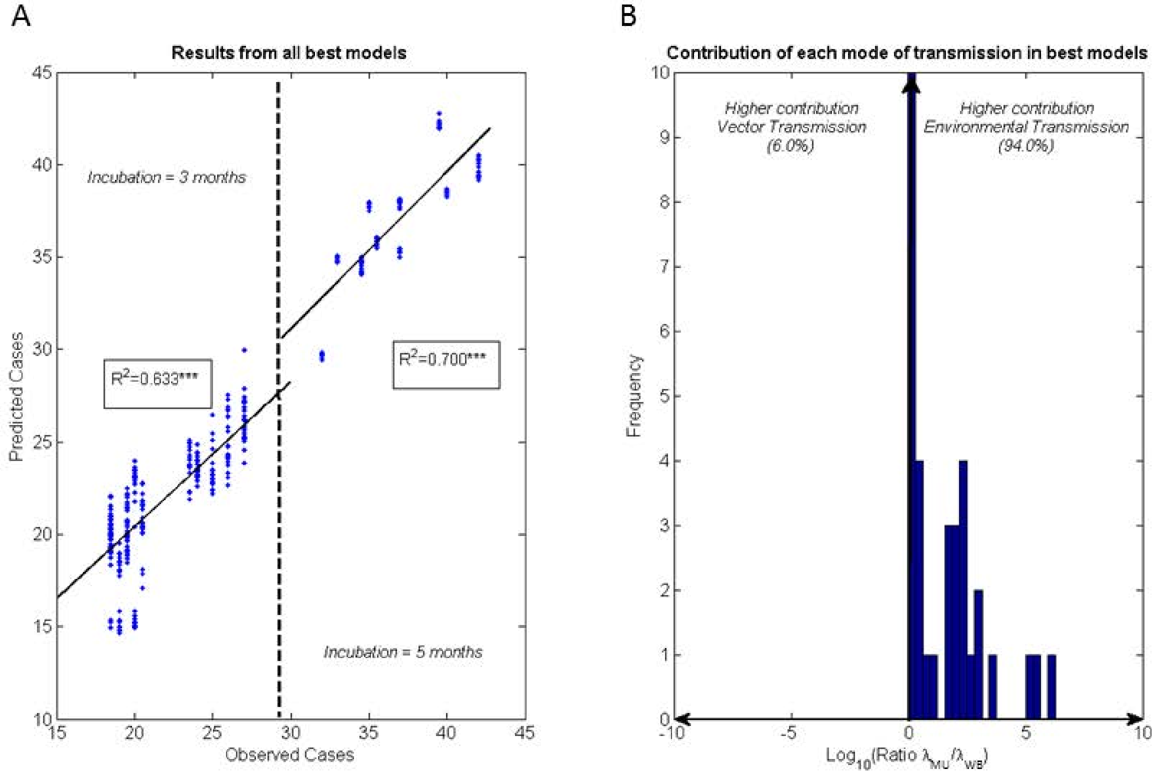

Figure S6. Predictions from the best set of fits in the mathematical model when considering times to seek treatment of 1-6 months.

A) Predictions from the set of best fits(AIC=[56.86-58.86], n=33) against the observed number of cases for an incubation period of 3 months (left) and 5 months (right). The solid black lines represent the linear regression line for each incubation period and the R-square of each regression is given in the inlet boxes (*** p<0.001).

B) Ratio of the mean force of infection from the environmental transmission over that from the vector transmission in the set of best fits. The ratio is in logarithmic scale (*i.e.* a ratio of 2 means that the environmental transmission was 100 times higher than the vector transmission). The vertical blue bars represent the number of fits with a certain ratio. In this set of fits, up to 6% of them predict a higher contribution of water bug transmission.

Table S3. Results from best temporal models including longer times to seek treatment (1-6months). Although the most consistent model fits with 6 months from exposure to treatment predict a much higher contribution of the environmental transmission, this contribution is reduced for longer times (8-9 months), where water bug transmission could contribute nearly as much.

Environmental Variable	Relationship	Time from Exposure to Treatment	Number of models	Mean λ_{MU}	Mean λ_{WB}	Mean AIC
MU_{con}	Linear	6	18	1.27E-04	1.13E-06	58.26
		8	3	8.95E-05	3.43E-05	58.51
		9	10	7.58E-05	5.12E-05	57.95
MU_{pos}	Linear	8	2	7.38E-05	4.97E-05	58.77

Section S8: Detailed results from statistical models of spatial distribution of BU

In the main text, a summary of the most relevant results from the statistical (spatial) models is provided. In this section, we explain in detail all the statistical models performed as well as the verification of model assumptions.

The results of univariate models show an association of BU incidence with variables suggestive of environmental transmission but not with the ones used as proxy of water bug transmission (Table S4 and Figure S7). Following this, we tested the improvement in model performance with bivariate analysis by adding a variable linked to water bug transmission to each of the environmental variables, but none of the bivariate models had a lower AIC than the univariate models considering only the environmental variables (Table S4).

General additive models applied to each of the environmental variables revealed different patterns for the link of BU incidence with *MU* concentration and *MU* positivity. First, the pattern for *MU* concentration in GAMs of different spans resembled slightly exponential (Figure S8), but the model performance for all spans tested (1-0.7) was progressively worse than the linear model (higher AIC). Second, the pattern for *MU* positivity approximated to a sigmoid function when reducing the span to values of 0.9 or lower (Figure S9). The GAM with a span value of 0.8 had the lowest AIC and performed significantly better than the linear model (Table S4). As a result we selected this model as the best performing spatial model (main text) and explored the model assumptions.

Table S4. Results from all statistical models. Results are divided in linear univariate analyses (top), bivariate and non-linear analyses (bottom), where *y* represents the mean BU incidence in a buffer, *a* is the intercept, *b* is the slope and *f* represents a non linear function. Only environmental variables were statistically associated with spatial patterns of observed cases.

Model	Model Formula	Span (LOESS smoother)	a	b1	b2	Defrees of freedom	AIC	F	Anova (Pr>F)
All Variables (linear univariate)									
LM	y=a+b1**MUcon*	-	0.69 *	0.32	-	29	80.87	-	-
	**y=a+b1*MUpos**	-	**0.82 *****	**5.00 ***	-	**29**	**78.96**	-	-
	y=a+b1**WBpos*	-	0.00 ***	0.77	-	29	83.79	-	-
	y=a+b1**WBinf*	-	0.00 ***	0.356	-	29	82.96	-	-
MU concentration									
**LM**	**y=a+b1*MUcon**	-	**0.69 ***	**0.32**	-	**29**	**80.87**	-	-
	y=a+b1**MUcon* + b2**WBpos*	-	0.73 *	0.37	-1.21	28	82.04	0.76	0.391
	y=a+b1**MUcon*+b2**WBinf*	-	0.63	0.30	0.01	28	82.2	0.45	0.442
GAM	y=a+f(*MUcon*) [1]	1	0.69	0.32	-	28.29	81.86	0.54	0.408
		0.9	0.69	0.32	-	27.81	82.84	0.32	0.618
		0.8	0.69	0.32	-	27.38	82.96	0.64	0.505
		0.7	0.69	0.32	-	26.78	83.73	0.63	0.557
MU Positivity									
LM	y=a+b1**MUpos*	-	0.82 ***	5.00 *	-	29	78.96	-	-
	y=a+b1**MUpos* + b2**WBpos*	-	0.9 ***	5.60 *	-1.26	28	79.98	0.90	0.349
	y=a+b1**MUpos* + b2**WBinf*	-	0.74 **	4.92 *	0.01	28	80.07	0.81	0.373
**GAM**	**y=a+f(MUpos)** [1]	1	0.82	5.00	-	28.06	79.54	1.26	0.266
		0.9	0.82	5.00	-	27.44	77.52	2.78	0.090
		**0.8**	**0.82 ***	**5.00 ***	-	**26.75**	**75.55**	**3.45**	**0.041**
		0.7	0.82	5.00	-	26.24	75.92	3.02	0.051

*p-value<0.05 ; ***p-value<0.001

Best statistical model for all variables (linear only), MU concentration, and MU positivity, is represented in bold

[1] The nature of the relationship using GAMs cannot be described with a single equation. See Figure S7 and Figure S8 for a graphical description of the relationship for each value of span. GAM of spans lower than 0.7 gave progressively higher AICs and are not included here.

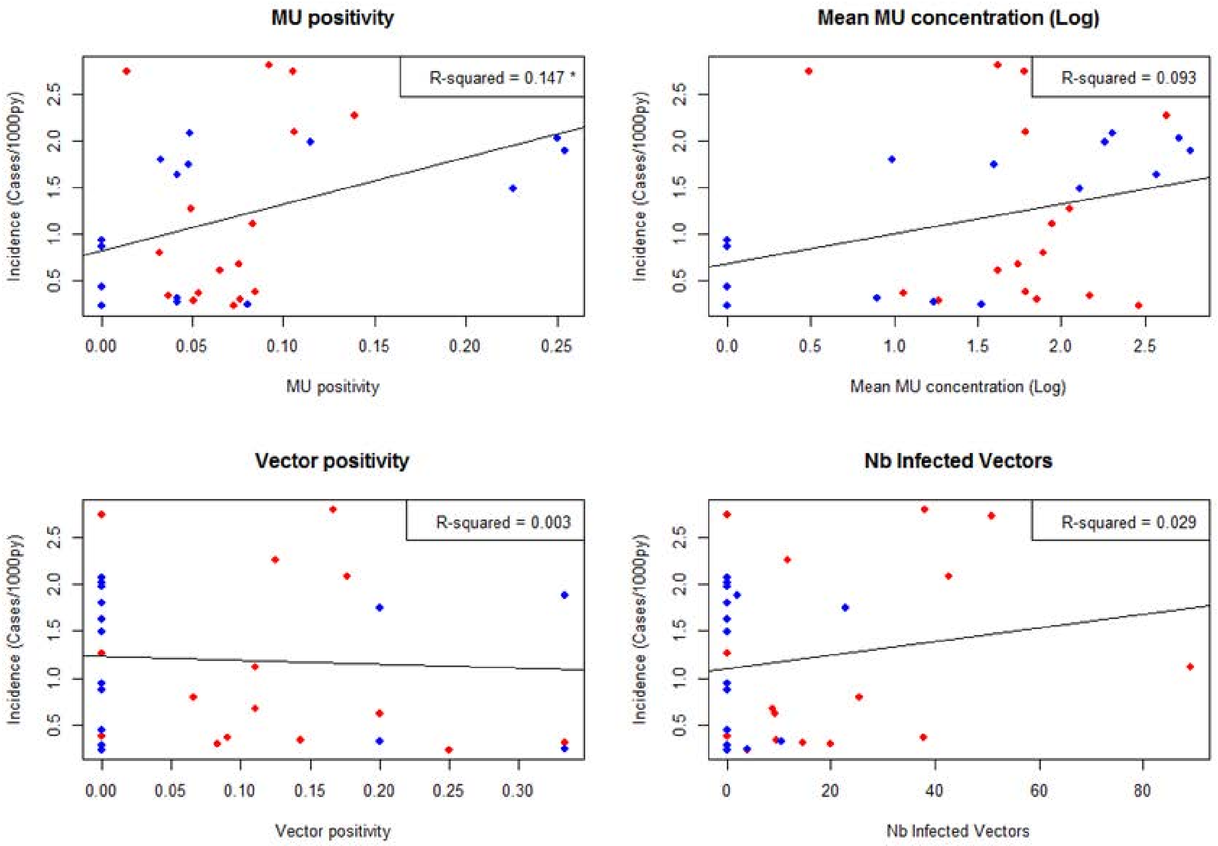

Figure S7. Univariate linear models for the spatial association of MU in the environment and vectors with Buruli ulcer. Red and blue dots represent the mean values for each site in Akonolinga and Bankim respectively. The solid line represents the linear fit, and the R^2 of each fit is given in the top right of the figure (* p<0.05). Only variables suggestive of environmental transmission (on top) presented signifficant associations, as opposed to water bug transmission (bottom)

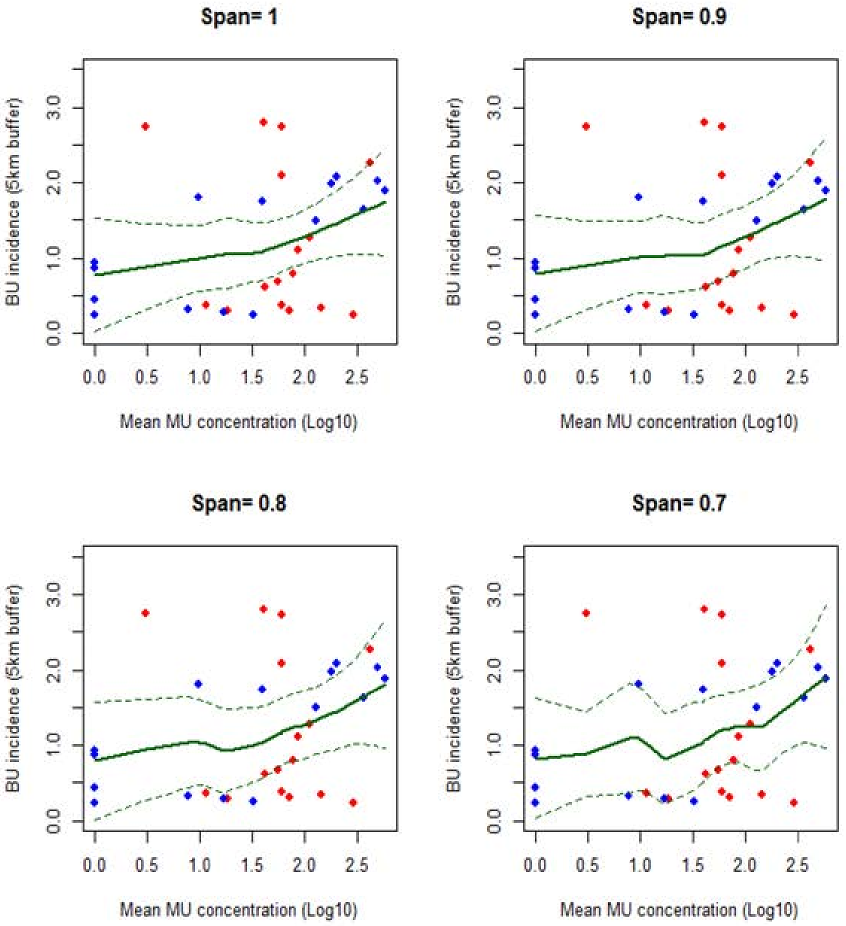

Figure S8. Univariate additive models for the spatial association of MU concentration in the environment with Buruli ulcer. Red and blue dots represent the mean values for each site in Akonolinga and Bankim respectively. The solid line represents the fit for each of the LOESS spans and the dashed lines are the 95% confidence intervals. None of these models improved the fit of the linear model.

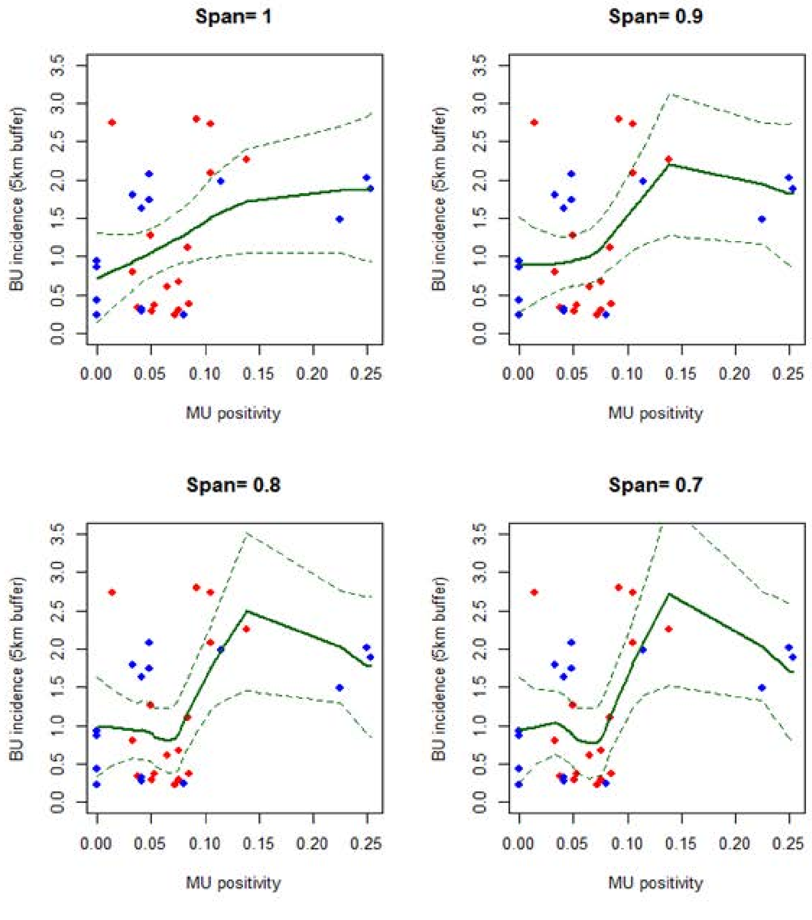

Figure S9 Univariate additive models for the spatial association of MU positivity in the environment with Buruli ulcer. Red and blue dots represent the mean values for each site in Akonolinga and Bankim respectively. The solid line represents the fit for each of the LOESS spans and the dashed lines are the 95% confidence intervals. The GAM with a Span=0.8 had the best fit and significantly improved the results of the linear model.

Normality of the residuals, homogeneity and independence were checked through exploration of standard model validation graphs. In addition, since we used data that was spread spatially across two regions, we checked for spatial correlation of the model residuals. For this, we plotted the spatial distribution of the residuals (through "gstat" and "sp" packages)[20,21] and we estimated the spline correlogram of the residuals for each region ("ncf" package)[22]. If model assumptions were violated, we explored other models that improved the distribution of the residuals and compared the results with those obtained with the original model.

The GAM model for *MU* positivity (span=0.8) revealed that residuals were not normal. In addition, spatial exploration of residuals for both regions revealed spatial patterns in their distribution. Incidence was underestimated at sites in the centre of Akonolinga and the south of Bankim and slightly overestimated in the rest of each region, and spatial autocorrelation in Akonolinga was significant at distances lower than 8.4km. For this reason, we applied a binomial GAM model with the same span using the incidence as a binomial variable in which the number of successes was the mean incidence in the buffer and this was weighted with the total population within the buffer. The binomial model largely improved the issues of the previous Gaussian model. The model residuals were normally distributed and spatial autocorrelation in Akonolinga was reduced. In addition, in order to check the influence of extreme values in the models, we performed a linear model using MU positivity as a dummy variable (cut-off at MU positivity 0.09 based on graphical exploration). The difference in BU incidence between high and low levels of *MU* positivity was highly significant ($p<0.001$), and in this model spatial autocorrelation was only significant for Akonolinga at distances smaller than 5km, which is the size of our spatial buffer.

The results of these models were qualitatively similar to the one included in the main text, and therefore we can conclude that the link between *MU* positivity and BU incidence spatially shows a sigmoid relationship with a threshold level and a saturation value.

Section S9: Exploration of model residuals in the best temporal model

We verified the normality of the residuals in the best temporal model to detect whether our prediction errors occured in a random fashion. A qq-plot of model residuals is shown in Figure S10 and suggests that the residuals were indeed normaly distributed, which further reinforce the results of our temporal model.

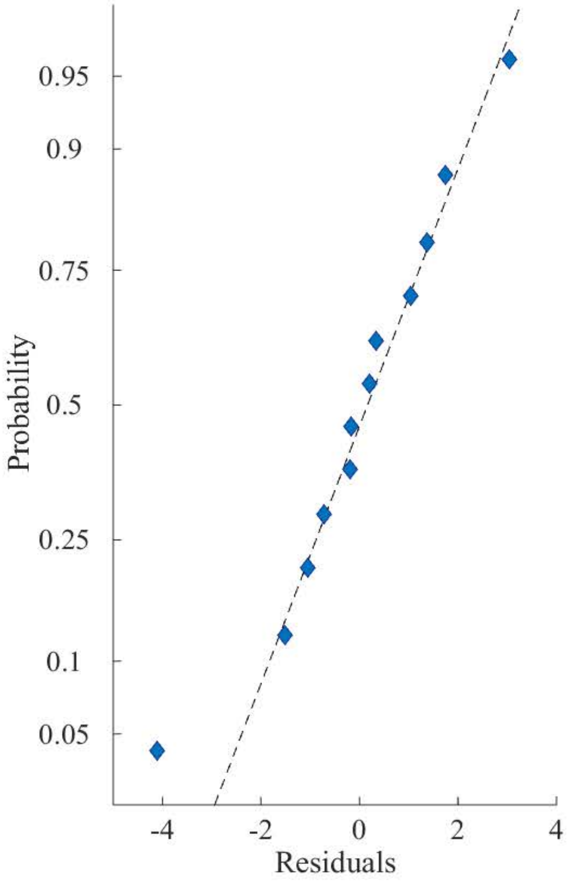

Figure S10. Quantile-Quantile plot of model residuals in the best temporal fit. Sample quantiles are compared to theoretical quantiles from a normal distribution. A linear pattern in the qq-plot suggest that the residuals are normally distributed.

References

1. Benbow, M. E. *et al.* Aquatic Invertebrates as Unlikely Vectors of Buruli Ulcer Disease. *Emerg. Infect. Dis.* **14,** 1247–1254 (2008).

2. Merritt, R. W. *et al.* Ecology and transmission of Buruli ulcer disease: a systematic review. *PLoS Negl. Trop. Dis.* **4,** e911 (2010).

3. Garchitorena, A. *et al.* Mycobacterium ulcerans Ecological Dynamics and Its Association with Freshwater Ecosystems and Aquatic Communities: Results from a 12-Month Environmental Survey in Cameroon. *PLoS Negl. Trop. Dis.* **8,** e2879 (2014).

4. Marion, E. *et al.* Seasonal and Regional Dynamics of M. ulcerans Transmission in Environmental Context: Deciphering the Role of Water Bugs as Hosts and Vectors. *PLoS Negl. Trop. Dis.* **4,** 10 (2010).

5. Marsollier, L. *et al.* Colonization of the salivary glands of Naucoris cimicoides by Mycobacterium ulcerans requires host plasmatocytes and a macrolide toxin, mycolactone. *Cell. Microbiol.* **7,** 935–43 (2005).

6. Marsollier, L. *et al.* Protection against Mycobacterium ulcerans lesion development by exposure to aquatic insect saliva. *PLoS Med.* **4,** e64 (2007).

7. Marsollier, L. *et al.* Early trafficking events of Mycobacterium ulcerans within Naucoris cimicoides. *Cell. Microbiol.* **9,** 347–55 (2007).

8. Marsollier, L. *et al.* Aquatic Insects as a Vector for Mycobacterium ulcerans. *Appl. Environ. Microbiol.* **68,** 4623–4628 (2002).

9. Anderson, R. M. & May, R. M. *Infectious diseases of humans: Dynamics and control*. (Oxford Science Publications, 1991).

10. R Development Core Team. *R: A language and environment for statistical computing*. (R Foundation for Statistical Computing, 2011).

11. Landier, J. *et al.* Spatio-temporal Patterns and Landscape-Associated Risk of Buruli Ulcer in Akonolinga, Cameroon. *PLoS Negl. Trop. Dis.* **8,** e3123 (2014).

12. Carolan, K. *et al.* Ecological niche modelling of Hemipteran insects in Cameroon; the paradox of a vector-borne transmission for Mycobacterium ulcerans, the causative agent of Buruli ulcer. *Int. J. Health Geogr.* **13,** 44 (2014).

13. Ravisse, P., Rocques, M. C., Le Bourthe, F., Tchuembou, C. J. & Menard, J. J. Une affection méconnue au Cameroun, l'ulcère à Mycobactérie. *Med Trop* **35,** 471–474 (1975).

14. Lipp, E., Huq, A. & Colwell, R. Effects of global climate on infectious disease: the cholera model. *Clin. Microbiol. Rev.* **15,** 757–770 (2002).

15. Roche, B. *et al.* Water-borne transmission drives avian influenza dynamics in wild birds: the case of the 2005-2006 epidemics in the Camargue area. *Infect. Genet. Evol.* **9,** 800–5 (2009).

16. Landier, J. *et al.* Seasonal patterns of Buruli ulcer incidence, Central Africa, 2002-2012. *Emerg. Infect. Dis.* **In press,** (2015).

17. Uganda Buruli Group. Epidemiology of Mycobacterium ulcerans infection (buruli ulcer) at Kinyara, Uganda. *Trans. R. Soc. Trop. Med. Hyg.* **65,** 763–775 (1971).

18. Trubiano, J. a, Lavender, C. J., Fyfe, J. a M., Bittmann, S. & Johnson, P. D. R. The incubation period of Buruli ulcer (Mycobacterium ulcerans infection). *PLoS Negl. Trop. Dis.* **7,** e2463 (2013).

19. Lavender, C. J. *et al.* Buruli ulcer disease in travelers and differentiation of Mycobacterium ulcerans strains from northern Australia. *J. Clin. Microbiol.* **50,** 3717–21 (2012).

20. Pebesma & J., E. Multivariable geostatistics in S: the gstat package. *Comput. Geosci.* **30,** 683–691 (2004).

21. Pebesma, E. & Bivand, R. Classes and methods for spatial data in R. *R News 5* **2,** 9–13 (2005).

22. Bjornstad, O. N. ncf: spatial nonparametric covariance functions. R package. (2013).
